# Community gender systems and a daughter’s risk of female genital mutilation/cutting: Multilevel findings from Egypt

**DOI:** 10.1371/journal.pone.0229917

**Published:** 2020-03-06

**Authors:** Kathryn M. Yount, Yuk Fai Cheong, Rose Grace Grose, Sarah R. Hayford

**Affiliations:** 1 Hubert Department of Global Health and Department of Sociology, Emory University, Atlanta, Georgia, United States of America; 2 Department of Psychology, Emory University, Atlanta, Georgia, United States of America; 3 Department of Community Health Education, Colorado School of Public Health, University of Northern Colorado, Greeley, Colorado, United States of America; 4 Department of Sociology, Ohio State University, Columbus, Ohio, United States of America; Washington University in St. Louis, UNITED STATES

## Abstract

We tested a feminist social-ecological model to understand community influences on daughters’ experience of female genital mutilation/cutting (FGMC) in Egypt, where over 90% of women ages 15–49 are cut. FGMC has potential adverse effects on demographic and health outcomes and has been defined as a human-rights violation. However, an integrated multilevel-level framework is lacking. We theorized that a more favorable *community-level gender system*, including stronger gender norms opposing FGMC *and* expanded extra-familial opportunities for women in the village or neighborhood, would be associated with a daughter’s lower risk of FGMC and would strengthen the negative association of a mother’s opposition to FGMC with her daughter’s risk of cutting. Using a national sample of 14,171 mother-daughter dyads from the 2014 Egypt Demographic and Health Survey, we estimated multilevel discrete-time hazard models to test these relationships. Community gender norms opposing FGMC had significant direct, negative associations with the hazard that a daughter was cut, but women’s opportunities outside the family did not. Maternal opposition to FGMC was negatively associated with cutting a daughter, and these associations were stronger where community opposition to FGMC and opportunities for women were greater. Results provided good support for a gender-systems framework of the multilevel influences on FGMC. Integrated, multilevel interventions that address gender norms about FGMC and structural opportunities for women in the community, as well as beliefs about the practice among the mothers of at-risk daughters, may be needed for sustainable declines in the practice.

## Introduction

*Female genital mutilation/cutting* (FGMC) refers to “procedures involving the partial or total removal of the external female genitalia or other injury to the female genital organs for non-medical reasons” [1]. An estimated 200 million women and girls in 30 countries have experienced FGMC [2], although estimates for the number affected vary [3]. On the African continent, FGMC is practiced in at least 29 countries, with estimated prevalences ranging from 1% in Cameroon and Uganda to 98% in Somalia [3]. The practice—and especially its more extensive forms–is associated with various health and demographic outcomes, including elevated risks of an earlier age at first sex [4], sexual trauma and dysfunction [5, 6], primary infertility [7], obstetric complications [8, 9], and poor marital outcomes [4, 8, 10]. The United Nations recognizes FGMC as a violation of human rights [1], and Sustainable Development Goal 5.3 establishes a global mandate to support abandonment of the practice [11]. Alongside legal and programmatic efforts to end FGMC [12], its prevalence has declined somewhat in more than half of 29 practicing African countries. Declines have been greater where prevalence has been low to moderate, but FGMC remains widespread in eight countries, including Egypt [13, 14].

Community context influences various outcomes in childhood and adolescence related to health [15, 16], sexual behavior [17], and harmful practices, such as (girl) child marriage [18]. Community characteristics also are associated with the practice of FGMC [19, 20]. Most of this research has focused on norms specific to FGMC—namely descriptive norms about what is practiced [19, 20]. Yet, norms about FGMC also include injunctive norms about what should be practiced [21], and FGMC norms are only one aspect of what we call a *community-level gender system*, which also includes structural opportunities for women outside the family.

We advance research on FGMC in three ways. First, we propose an explanatory model that identifies two central elements of a *community-level gender system*: the collective practice of FGMC as a *gender norm* that upholds patriarchal family systems, and the *structural opportunities for women* outside this family system. Identifying *both* characteristics as central elements of a community-level gender system debunks debates about normative *versus* structural drivers of demographic and behavioral change [22, 23], and instead, highlights their joint influences [24], especially for outcomes that tend to disadvantage girls, such as skewed sex ratios at birth [25], excess mortality in childhood [26, 27], and child marriage before age 18 [28]. Second, we apply rigorous multilevel analytical methods to assess how both of these elements of a community-level gender system are associated with the risk that a daughter is cut, directly and by moderating the influence of a mother’s FGMC attitudes and experience. Third, we focus on the setting of Egypt, where FGMC remains widespread, and a majority of women favor continuation, despite criminalization of the practice. In 1995, 97% of ever-married women 15–49 years were cut [29–32]. Since FGMC was criminalized in 2008, most girls 15–19 years continue to be cut (88% in 2014 vs. 94% in 2008) [33], and the majority of ever-married women 15–49 years support continuation (58% in 2014 vs. 62% in 2008) [33]. Thus, this study fills conceptual and empirical gaps in research on the multilevel influences on FGMC in the so-called classic patriarchal belt of the Middle East and North Africa (MENA). Our framework and findings offer insights for multifaceted, multilevel interventions to further the abandonment of FGMC, and other practices harmful to women and girls.

## Background

### Determinants of FGMC

Studies of the determinants of FGMC have largely focused on family or household characteristics—a girl’s immediate social context. These studies have shown, across diverse settings, that daughters who are higher birth order [34], have mothers with more schooling [34, 35], and live in wealthier households [13, 36] less often have been cut. Religious also affiliation is associated with experience of FGMC, although the strength and direction of this association varies across contexts [20, 34, 37].

To date, few studies have examined the community-level influences on FGMC. Important identified influences have included the extent of ethnic diversity in the village or neighborhood [38], social networks among women [39], and local marriage markets [10, 19, 20, 34, 37–39]. Such evidence suggests that girls are embedded in multiple “communities” that may or may not be geographically bounded and may vary in the nature and composition of social ties. In research on FGMC, however, even neighboring villages can harbor different FGMC-related attitudes and practices, suggesting an important potential influence of village or neighborhood context on girls’ experiences [40].

In Egypt, one’s village or neighborhood of residence is salient for FGMC outcomes because *patrilocal endogamy* is common [41]; specifically, young women tend to marry biological relatives and to live with their husband’s family in close proximity to their birth family. As a result, extended patriarchal kinship structures cluster geographically in ways that influence collective practices like FGMC and local opportunity structures for women outside the family. For these reasons, we rely on the work of Macintyre and colleagues [42] to define “community” as the shared norms and opportunity structures that characterize one’s (village or) neighborhood of residence.

In the first multilevel analyses of FGMC, the village or neighborhood prevalence of FGMC was directly, positively associated with a girl’s risk of cutting in Kenya [19] and modified the association of household religious identity with this risk in Burkina Faso [20]. FGMC also tends to be less common in urban than rural areas [13]; however, such differences may be reduced or reversed after accounting for other factors [19, 20, 34]. The influences of gender-related village or neighborhood characteristics on a daughter’s risk of FGMC are undertheorized and understudied.

### Community gender systems and FGMC: A feminist framework integrating social norms and structural opportunities

**Fig 1:**
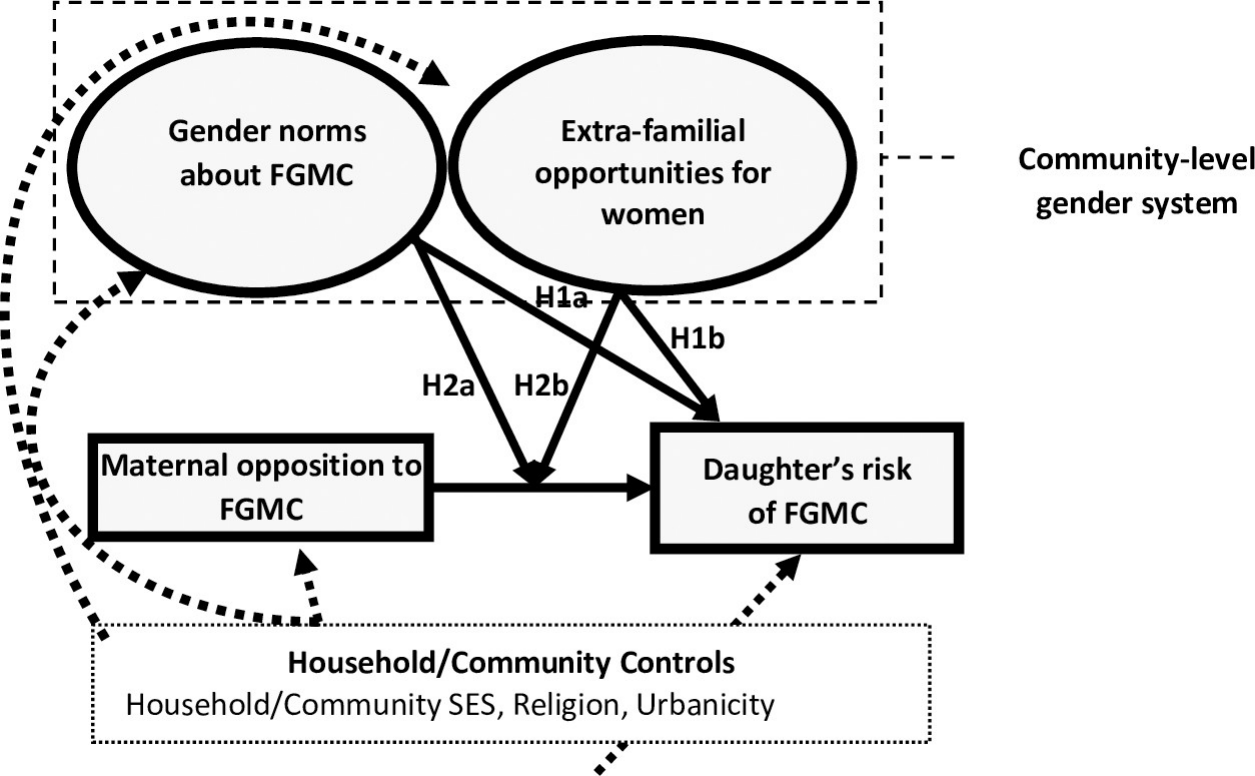
Multilevel influences on FGMC: Conceptual model. Solid arrows denote relationships of interest.

We propose an integrated, feminist, social-ecological framework to understand how gender-related village or neighborhood characteristics may influence the risk of FGMC (Fig 1). Specifically, we argue that a *community-level gender system* consists of *gender norms about FGMC* and *extra-familial opportunity structures that are more or less open to women*. These elements of the local (village- or neighborhood-level) gender system are expected to influence a daughter’s risk of FGMC, directly and by conditioning the influence of maternal beliefs about and personal experience with the practice.

#### Community gender norms about FGMC

A *social norm* is a collective practice in a referent group that is motivated by two *social expectations* in the group: the *empirical expectation* that others practice the behavior, and the *normative expectation* that others think the practice should be upheld [21, 43–45]. A social norm is maintained through social influence, or expectations that compliance will be rewarded with social acceptance, while non-compliance will be punished with social rejection [45]. Thus, complying with a social norm can be a marker of social belonging [46–48], and sustaining the norm serves to uphold these social ties as distinct and valued [37].

In the case of FGMC, social norms theorists argue that the decision to cut or not to cut a daughter will depend on parental perceptions of the collective practice in their community and of the community’s shared expectations about it [46, 47]. A mother (usually the parent most directly involved in decisions about FGMC) may believe personally that FGMC is unnecessary or even harmful; however, she may have her daughter cut if she perceives in her community that: FGMC is widely practiced (*descriptive norms* are high), others feel strongly that FGMC should be practiced (*injunctive norms* are high), and non-compliance will have negative social consequences for her daughter. That said, this mother may not have her daughter cut if she perceives that her uncut daughter will be socially accepted in the community. Social norms theorists, therefore, hypothesize an interaction between personal beliefs and community norms about a practice. In communities where the social norm is to strongly favor FGMC, opposing personal beliefs are expected to have little influence on behavior. As this social norm weakens, a “tipping point” is expected to be reached, where the influence of personal opposition to FGMC can be observed [46].

Social norms approaches to FGMC have some empirical support. The community-level prevalence of FGMC, a proxy for *descriptive norms* about the practice, has been positively associated with the risk of cutting a daughter in parts of Africa [19, 20]. In communities in Egypt and parts of Africa, public denunciations of FGMC have signaled changes in *injunctive norms* about the practice and have been thought to spur declines in prevalence [13, 49, 50]. Such evidence suggests that community-level descriptive norms and injunctive norms about FGMC matter; however, their joint influences are understudied, including whether a tipping point in community opposition to FGMC is needed for maternal opposition to have an influence on a daughter’s risk of cutting [51, 52].

A limitation of social norms theory is its cursory discussion of FGMC as integral to broader norms about womanhood. In many practicing societies, FGMC is performed to reflect and to uphold injunctive norms about sex differentiation, feminine beauty, and women’s sexuality [5, 10, 53, 54]. First, women achieve social recognition by becoming less like men physically [53]; thus, by removing the “male parts” and sometimes enclosing the “female parts,” FGMC “feminizes” a girl’s body [53–55]. Second, FGMC helps to realize the feminine aesthetics of purity, smoothness, and cleanliness [53, 55]. Third, by de-emphasizing (and controlling) women’s sexuality, FGMC accentuates women’s reproductive role [53]. In these ways, the collective practice of FGMC acts as a *gender norm*, reinforcing the injunctive norm that *good—physically appealing and sexually modest—women* are cut, and therefore, must be cut to be marriageable in a patriarchal family system.

In Egypt, FGMC indeed is closely tied to expectations about gender identification, feminine beauty and purity, and the control of women’s sexuality [34, 56–59]. The extended family is a core social institution that has strong geographic affiliations and interests that often override those of an individual. Patriarchal control and family honor are constructed and sustained through the common practices of endogamous, patrilocal marriage and the social and sexual control of women. By ensuring a girl’s virginity and conformity to expectations of feminine modesty and aesthetics, FGMC is thought to enhance a daughter’s prospects for a suitable marriage, often within the extended kin group [34]. This way, FGMC operates as a gender norm that reflects and reinforces what Kandiyoti [60] has called a classic patriarchal family system.

In recent decades, minority Christian groups in Egypt have begun to abandon FGMC while Islamist groups have promoted FGMC as part of authentic Muslim womanhood [37]. Thus, higher percentages of Muslim than Christian women practice FGMC, intend to cut their daughters, and view FGMC as desired by religion, “good” for the girl, cleansing or purifying, and protective against sexual transgressions [34]. Findings from the 2014 Survey of Young People in Egypt confirm that Christian women and men are less likely than Muslim women and men to report favorable views about the practice [61]. Thus, the emergence in Egypt of FGMC as a symbol of authentic Muslim womanhood has transformed the practice from a widespread gender norm to a gender norm that defines Muslim identity [37]. The prevalence of FGMC (97% rural South; 86% urban North) and endorsement of continuation (73% rural South; 38% urban governorates) also vary substantially geographically, with the gender norm of FGMC being strongest in the rural South.

#### Community structural opportunities for women outside the family

The gender norm of FGMC may exist alongside *structural inequities* that limit women’s opportunities outside the patriarchal family [35, 62–64]. These structural inequities are multifaceted, arising in social, economic, and political spaces [65]. Social inequities manifest in women’s lack of access to (especially higher) education and to the means by which they can enact their sexual and reproductive choices. Economic inequities manifest in women’s lack of access to activities and institutions involving the production, distribution, and consumption of goods and services [66]. Political inequities manifest in women’s lower participation in politics and lower representation at various levels of government [67]. Together, these structural barriers limit women’s access to resources and identities outside the family, cementing women’s dependence on (male) family members and women’s conformity to practices that uphold this family system. In lower- and middle-income settings, evidence of structural gender equities at the village or neighborhood level may include higher average schooling attainments, later average ages at first marriage, lower rates of arranged marriage, and higher rates of participation in market work. The availability of these opportunities in a mother’s local community (village or neighborhood) may allow her to see real alternatives for social acceptance instead of having her daughters cut [35, 44, 68]. Thus, a direct association may exist between (proximate) community opportunities for women outside the family and a daughter’s risk of FGMC.

Social norms theorists have questioned the influence of women’s extra-familial opportunities on the risk of FGMC, citing patriarchal settings where FGMC is not practiced and women’s involvement in its continuation [69]. These arguments ignore feminist theory [60]. First, different forms of patriarchy exist [60] and are upheld by gender norms that may or may not include FGMC. Second, women’s complicity in upholding harmful practices may reflect a survival strategy under patriarchy [60], where mothers pursue the best available “options” for their daughters in the absence of real alternatives. Thus, the expansion of local opportunities for women outside the family but in the village or neighborhood may signal the availability of real alternatives for women, thereby reducing the perceived costs of violating gender norms favoring FGMC. This perspective implies an interaction between maternal opposition to FGMC and the local structure of opportunities for women outside the family. If local opportunities for women are limited, a mother who opposes FGMC may not see real alternatives for her daughter and may have her daughter cut anyway as the best “option” to secure her future; however, this mother may be willing to leave her daughter uncut of opportunities for women’s education, later marriage, and work are apparent in her village or neighborhood.

In Egypt, opportunities for women outside the family remain limited, but geographic variation in these opportunities is substantial. With respect to marriage, more than 17% of women 20–24 years are married as children (before 18 years) nationally; however, the median age at first marriage varies from 19.1 years in the rural South to 23.1 years in the urban governorates [70]. Nationally, one third of ever-married women (regardless of age) report consanguineous (or blood) marriage, but the rates of such marriages vary from 49% in the rural South to 19% in the urban North [70]. Women’s economic dependence on the family also remains high, as evidenced by somewhat lower rates than men of high school completion (33% vs. 38%) [70] and much lower rates than men of post-marital market work (18% vs. 98%) [71, 72]. Geographically, ever-married women’s disadvantage in completed secondary schooling is highest in the rural South (20% vs. 30% for men) and lowest in the urban North (45% vs. 46% for men), and women’s low rates of market work vary similarly, from 11% in the rural South to 22% in the urban North [70].

### Summary and hypotheses

In sum, an integrated, feminist, social-ecological framework situates the decision to cut or not to cut a daughter within a *community gender system*. In Egypt, this system is aptly measured at the village or neighborhood level because marriage within the kin group and co-residence are high. This system includes *gender norms* about FGMC and *extra-familial opportunities* available or unavailable to women. Both of these elements of a community gender system are expected to influence *directly* a daughter’s risk of FGMC and to *condition* the influence of maternal opposition to FGMC on this risk. Specifically:

*H1*. A daughter will have a lower adjusted hazard of FGMC in communities *(a)* that have a lower prevalence of FGMC and more strongly oppose the practice, and *(b)* where women have more structural opportunities outside the family.
*H2*. A mother’s opposition to FGMC and non-cut status will be associated with a lower hazard of her daughter being cut in communities *(a)* that have a lower prevalence of FGMC and more strongly oppose the practice, and *(b)* where women have more structural opportunities outside the family. In communities that have a higher FGMC prevalence and more strongly favor the practice and where women have fewer structural opportunities outside the family, maternal opposition to FGMC will not be associated with a daughter’s risk of FGMC.

## Method

### Sample and data

The data source for this analysis was the 2014 Egypt Demographic and Health Survey (DHS). All DHS data are publicly available from Measure DHS to registered users (https://dhsprogram.com/data/new-user-registration.cfm). The DHS gathers nationally representative data from women of reproductive age and their households on household socioeconomic conditions, women’s marriage and birth histories, schooling and work, and FGMC, among other data. The DHS typically use a two-or three-stage probability sampling design, in which small geographic areas are selected, followed by households and respondents within households. These small-sample areas are effective proxies for villages or urban neighborhoods, salient “communities” in Egypt [73].

The 2014 EDHS included 883 sample clusters after excluding 42 other clusters in Sinai (<1%) for security reasons. On average, 25 households per cluster were sampled. Within each household, all ever-married women 15–49 years were interviewed, for a total sample size of 21,762 women. Because our outcome was the FGMC experience of daughters, we treated the mother-daughter dyad as the individual-level, or level-one, unit of analysis. The sample cluster was the community-level, or level-two, unit of analysis. For mothers with more than one daughter, we randomly selected one daughter for the analysis. Women with no daughters were included only in constructing level-two measures (see below). After dropping mother-daughter dyads with missing data (*n* = 26), the final sample size was 14,171 mother-daughter dyads within households nested in 881 communities. On average, these clusters included 16 mother-daughter dyads (ranging from 1 to 78 dyads across clusters, with a standard deviation of 8). Less than 1% of clusters included 1 one woman, 7.5% had 5 or fewer women, and 27% had 10 or fewer women. For primary exposure variables, the community-level sample averaged 24 ever-married women 20–49 years per cluster (ranging from 1 to 127 across clusters, with a standard deviation of 13).

The 2014 EDHS collected three general types of data on FGMC for all women respondents and their daughters [14, 70]. All ever-married women 15–49 years self-reported their experience with FGMC, including type and timing, and their own attitudes about FGMC. All women in the sample with daughters exact age 20 or younger reported their daughters’ experiences with FGMC, including age of the event for cut daughters.

### Main variables

#### Outcome: Daughter’s FGMC status

Our outcome was the *daughter’s experience of FGMC*. For each daughter aged 20 years or younger, the mother reported whether her daughter was cut. If the response was “yes,” the mother reported the daughter’s age at cutting in years.

#### Individual (level-one) exposure

A measure for *maternal opposition to FGMC* was derived from a factor analysis of four items. The first item captured whether the mother reported to be *uncut* (0 = no, 1 = yes). Inclusion of cutting status was justified conceptually and empirically. First, the cut or uncut status of the mother arguably captured a preference for the practice among her own parents [74, 75]. Second, evidence from Egypt has shown a strong, positive association of maternal FGMC status with maternal intent to cut a daughter and a daughter’s risk of being cut [34]. We treated this item as dichotomous because self-reports of any versus no cutting are reasonably reliable [29, 76, 77]. The second and third items captured whether the mother believed FGMC was *not* required by religion (0 = no, 1 = yes) and should *not* continue (0 = no, 1 = yes). For both questions, the original response option ‘don’t know’ was recoded as 0 to distinguish clearly unfavorable attitudes about FGMC. Because of the strong association of Christian religion with unfavorable attitudes about FGMC and non-cutting in Egypt [34, 37], we added a fourth item for whether the mother was Christian (= 1) or Muslim (= 0). In random split-half samples, we used exploratory factor analysis (EFA) and then confirmatory factor analysis (CFA) to assess whether these items reflected a unidimensional construct. A single-factor CFA model with all four items had an adequate fit to the data (factor loadings 0.53–0.98, Root Mean Squared Error of Approximation [RMSEA] = 0.02, Comparative Fit Index [CFI] = 0.99, Tucker-Lewis Index [TLI] = 0.99, α = 0.65) [78–80].

#### Community (level-two) exposures

We considered four items to capture *community-level gender norms opposing FGMC*. Items were created using data from all ever-married women 20–49 years in the community, a separate, non-overlapping, cohort from the daughters ages 0–20 on which the outcome was observed. One measure of descriptive norms about FGMC was the proportion of women in the community who were *uncut*. Two measures of injunctive norms about FGMC were the proportions of women agreeing that religion does *not* require FGMC and that it should *not* continue. The proportion of women who were Christians was considered for the reasons described above. In random split-half samples of communities, we performed EFA and then CFA to assess whether these four items reflected a unidimensional construct. The proportion Christian did not adequately load and was dropped. A single-factor CFA model with the remaining three items [81] had an adequate fit to the data (factor loadings 0.72–0.99, RMSEA = 0.12, CFI = 0.93, TLI = 0.90, α = 0.89). Although the 2014 EDHS had a men’s sample, we used women’s responses to capture community gender norms about FGMC because men often report less favorable attitudes about FGMC than do women [82, 83].

We also created a factor score for *women’s community-level structural opportunities outside the family*. This score was based on the proportions of ever-married women 20–49 years in the community who had completed secondary school or higher, had first lived with a spouse at age 18 or older, had married a non-cousin, and had worked for cash or kind in the prior year. We considered adding a measure for women’s ownership of land, but less than two percent of ever-married women 15–49 years owned land in 2014 [84]. We also considered aggregate scores for women’s freedom of movement and influence in household decisions; however, questions on freedom of movement were not asked in the EDHS 2014, and measures for freedom of movement and decision-making capture women’s agency in the marital family [64], not opportunities outside the family, a core element of our theory. Also, in Egypt, women’s age at first marriage, schooling attainment, and market work are determinants of their agency in the marital family [68]. We performed EFA and then CFA in random split-half samples. A single-factor CFA model with all four items had an adequate fit to the data (factor loadings 0.57–0.84, RMSEA = 0.12, CFI = 0.93, TLI = 0.90, α = 0.68).

#### Individual (level-one) covariates

We controlled for mother-daughter characteristics that have been associated with a daughter’s risk of experiencing FGMC. We included the daughter’s birth year in calendar years to control for cohort differences in FGMC risk [33], daughter’s birth order among daughters [34], mother’s age in years [20, 34], father’s schooling attainment (1 = completed secondary or higher, 0 = less than completed secondary) [34, 85], and a DHS-created household wealth score dichotomized to capture the wealthiest two quintiles (top 40%) versus the bottom 60% of households [3, 34].

We also controlled for a composite level-one measure for maternal opportunities. This measure was derived from a factor analysis of four items: maternal reported age at first residence with a spouse (0 = 17 or younger, 1 = 18 or older), non-cousin marriage (0 = no, 1 = yes), completed secondary school or more (0 = no, 1 = yes), and work outside the home for cash or kind in the prior year (0 = no, 1 = yes). In random split-half samples, we performed EFA and then CFA to assess whether these items reflected a unidimensional construct. Non-cousin marriage loaded poorly and was dropped. A single-factor CFA model with the remaining three items had an adequate fit to the data (factor loadings 0.40–0.99, RMSEA = 0.02, CFI = 0.99, TLI = 0.99, α = 0.48).

#### Community (level-two) covariates

We controlled for three community-level characteristics that could confound the relationship between community FGMC norms, women’s structural opportunities outside the family, and a daughter’s risk of FGMC: the proportion of households in the wealthiest two quintiles, defined above [19]; urbanicity using DHS definitions for urban versus rural locality [13, 34]; and the proportion of ever-married women 15–49 years who were Muslim [13, 37]. All ever-married women 15–49 years were used to construct the last two variables to control for a community’s religious and urban composition with all available data.

#### Analyses

The analysis was of de-identified secondary data, so the study was exempt from IRB review. We began with descriptive analyses showing the characteristics of mother-daughter units and variation in the characteristics of these units according to community-level opposition to FGMC. All level-one descriptive statistics were weighted, and robust standard errors were estimated to account for the stratified, cluster-sample design. We also presented point estimates and ranges for community-level characteristics (unweighted because the DHS does not provide cluster-level weights).

We used multilevel discrete-time hazard models to estimate associations of a daughter’s hazard of FGMC with factor scores for maternal FGMC attitudes and status, community FGMC norms, and community opportunities for women [86–88]. In general, hazard models predict the likelihood of an event (FGMC) at each age, accounting for the possibility that girls who are uncut at the time of the survey may be cut in the future. To facilitate model estimation, we used age-risk-sets, or multi-year periods of exposure, rather than person-years of exposure. The multilevel structure of the models accommodated the hierarchical nature of the data and avoided the problem of biased estimates of precision that may result from ignoring clustering within communities. All models were estimated using STATA 14.1 [89].

Let *η*_*ijt*_ denote the log-odds of the baseline hazard of FGMC for the daughter of mother *i* living in community *j* at daughter’s age risk set *t*. Let *A*_*ijt*_ denote the daughter’s age risk set, specified as T1 = 0–1 years, T2 = 2–11 years, T3 = 12–16 years, and T4 = 17–20 years to capture major age-specific risk sets for FGMC in this setting [86–88, 90]. Since the model estimated was a limited dependent variable hazard model, we included all four age risk sets capturing ages 0–20 to provide maximum information to estimate the hazard for each age risk set. Although the median age of cutting in 2014 in Egypt was 10.5 years among ever-married women ages 15–49 [84], the probabilities of cutting at individual ages before age 10 ranged from .01 to .05 and were substantial enough that we did not truncate the sample of girls to those ages 10 to 20. Let *FGMC*_*ij*_ denote the mother’s FGMC attitudes and status, *N*_*j*_ the community FGMC norms, and *O*_*j*_ the community extra-familial opportunities for women. A general two-level discrete-time hazard model can be depicted as:

$$\eta_{ijt}=\sum_{t=0}^{T}\gamma_{t}A_{ijt}+\gamma_{F}FGMC_{ij}+\gamma_{N}N_{j}+\gamma_{O}O_{j}+\gamma_{FN}FGMC_{ij}*N_{j}+\gamma_{FO}FGMC_{ij}*O_{j}+u_{j}$$ (1)

Eq (1) shows *η*_*ijt*_ as a function of the age risk set *t* of the daughter of mother *i* in community *j*; main exposure variables at level-one and level-two and their interactions; and random community-specific contributions, *u*_*j*_, which are assumed to be normally distributed with variance δ. The γ_t_’s identify the logit hazard curve, and γ_*F*_, γ_*N*_, γ_*O*_, γ_*FN*_, and γ_*FO*_, identify the effects associated with the exposures and their interactions. Controls at level-one and level-two are added to Eq (1) but are not interpreted [91]. Level-one variables were cluster-mean centered, and level-two variables were grand-mean centered. The magnitude and significance of coefficients for level-two exposures (γ_*N*_, γ_*O*_) provided tests of hypothesis 1, regarding direct community effects. The magnitude and significance of coefficients for the interaction terms (γ_*FN*_, γ_*FO*_) provided tests of hypothesis 2, regarding the influence of maternal opposition to FGMC, conditional on the community-level gender system.

To assess the “tipping point” hypothesis of social norms theory [46, 47, 92], significant cross-level interactions were probed to detect the range of values for the community exposures for which maternal opposition to FGMC was significantly associated with her daughter’s FGMC risk [93]. When community *support* for FGMC is high, maternal opposition to FGMC is not expected to be protective; however, when community *opposition* to FGMC is high, and a new, self-sustaining norm *against* FGMC is apparent, the protective influence of maternal opposition to FGMC is expected to be observed. The “tipping point” refers to the boundary levels of community opposition to FGMC or community opportunities for women at which maternal opposition to FGMC is significantly negatively related to a daughter’s hazard of FGMC.

To assess the proportional odds assumption of these models, that all covariates have the same effects for all age risk sets [90], we tested the significance of two-way interactions of all covariates with the age-risk-set indicators (available on request). Our final models controlled for two covariates with non-proportional effects, maternal opposition to FGMC and daughter’s birth year, by retaining their interaction terms in the final model. For Muslim and living in an urban area, we attempted to test the proportional odds assumption that the logit-hazard curves of the communities were parallel to one another, holding constant all predictors [90]; however, the algorithm could not produce estimates, perhaps because less than 1% of observations on these two variables were in the last age risk set.

We did not apply multilevel sampling weights to account for the unequal probabilities of cluster and participant selection because the DHS does not provide cluster-level sampling weights [94]. In a similar single-level analysis that incorporated individual-level sampling weights and stratification information, inferences were robust to the inclusion or exclusion of weights.

## Results

### Characteristics of the mother-daughter sample

On average, daughters were eight years old at the time of the survey (born in 2005), and either the first- or second-born daughter (Table 1). At the time of the survey, 20% of daughters had been cut, and, as expected, this prevalence was monotonically higher across age groups of daughters, with less than 1% of 0–1-year-olds, 6% of 2–11-year-olds, 49% of 12–16-year-olds, and 65% of 17–20-year-olds cut.

**Table 1:** Characteristics of ever-married mothers 15–49 years and their daughters 0–20 years, 2014 Egypt Demographic and Health Survey (*N* = 14,171 mother-daughter dyads).

				Distribution of mother-daughter dyads by community % of ever-married women 20–49 years saying FGMC should NOT continue		
	Full Sample			Lowest tertile of community opposition (*n* = 4,628)	Middle tertile of community support (*n* = 4,802)	Highest tertile of community opposition (n = 4,741)
	*M*	*SD*	Range	*M*	*M*	*M*
**Daughter's Characteristics**						
Daughter experienced FGMC	.20	.40	0 to 1	.35	.21	.07
Daughter 0–1 experienced FGMC	.002	.04	0 to 1	.03	.01	.00
Daughter 2–11 experienced FGMC	.06	.24	0 to 1	.22	.06	.01
Daughter 12–16 experienced FGMC	.49	.50	0 to 1	.63	.36	.10
Daughter 17–20 experienced FGMC	.65	.47	0 to 1	.89	.66	.27
Age in Years	7.91	5.75	0 to 20	7.84	7.79	7.97
Birth Year	2005.49	5.75	1994 to 2014	2005.58	2005.60	2005.15
Birth Order among Daughters	1.31	0.61	1 to 6	1.36	1.30	1.30
**Maternal FGMC Status and Attitudes about FGMC**						
Mother Experienced FGMC	.93	.25	0 to 1	.99	.96	.77
FGMC Required by Religion						
Yes	.53	.50	0 to 1	.70	.54	.28
No	.30	.46	0 to 1	.17	.27	.53
Don't Know	.18	.38	0 to 1	.13	.19	.20
FGMC Should Continue						
Yes	.60	.49	0 to 1	.85	.61	.26
No	.30	.46	0 to 1	.11	.27	.58
Don't Know	.10	.30	0 to 1	.04	.11	.15
**Maternal Demographic Characteristics**						
Age in Years	33.60	7.53	15 to 49	33.38	33.63	34.25
Married	.95	.21	0 to 1	.96	.95	.95
Religion is Muslim	.96	.19	0 to 1	.98	.96	.95
Household Wealth						
Poorest	.19	.39	0 to 1	.32	.18	.08
Poorer	.20	.40	0 to 1	.30	.19	.08
Middle	.22	.41	0 to 1	.21	.22	.13
Richer	.21	.40	0 to 1	.12	.24	.25
Richest	.19	.39	0 to 1	.05	.18	.47
Living in Urban Area	.35	.48	0 to 1	.18	.40	.72
Husband Completed Secondary School or Higher	.56	.50	0 to 1	.53	.56	.65
**Maternal Extra-Familial Opportunities**						
Completed Secondary School or Higher	.52	.50	0 to 1	.42	.52	.67
Age at First Spousal Coresidence 18+	.82	.39	0 to 1	.73	.84	.88
No Cousin Marriage	.67	.47	0 to 1	.54	.67	.77
Worked for Cash or Kind	.15	.35	0 to 1	.11	.16	.18
**Factor Scores**						
Maternal Opposition to FGMC	.09	.57	-.49 to 1.86	-.16	.05	.48
Maternal Extra-Familial Opportunities	-.08	.49	-1.02 to 1.13	-.23	-.07	.10

All means, proportions, and SDs are weighted.

On average, mothers were 34 years old, married (95%), Muslim (96%), living in a rural area (65%), and married to a spouse with completed secondary schooling (56%) (Table 1). A majority of mothers had completed secondary school (52%) and were age 18 or older when they began living with their spouse (82%). Only 15% had worked for cash or kind in the prior year. All mothers had heard of FGMC, most were cut (93%), and a majority felt FGMC was required by religion (53%) and should continue (60%).

The characteristics of daughters and mothers differed across communities with the lowest, middle, and highest opposition to FGMC, measured as tertiles of the community proportion of ever-married women 20–49 years believing that FGMC should not continue (Table 1). Only 7% of daughters were cut in communities with the highest opposition to FGMC, compared to 21% and 35% of daughters being cut in communities where opposition to FGMC was moderate or low, respectively. Also, the percentages of mothers who were cut (99% to 96% to 77%), believed that FGMC was required by religion (70% to 54% to 28%), and believed that FGMC should continue (85% to 61% to 26%) were monotonically lower with higher community opposition to FGMC (Table 1). Maternal secondary schooling, work for cash or kind, and first spousal co-residence at age 18 or older were more common in communities with high opposition to FGMC than in communities with low or moderate opposition to FGMC. Mothers whose spouse had the least schooling, who lived in the poorest households, and who lived in rural areas tended to live in communities with the lowest opposition to FGMC (Table 1).

### Characteristics of communities

About half of communities (51%) were urban (Table 2). In the average community, a majority of women were Muslim (96%), first lived at age 18 or older with a spouse (85%), married a non-cousin (68%), had completed secondary school (57%), and lived in the wealthiest 40% of households (52%); however, a minority of women had worked for cash or kind in the prior year (16%), were uncut (11%), believed that FGMC should stop (36%), and believed that religion did not require FGMC (34%).

**Table 2:** Level-two community characteristics, 2014 Egypt Demographic and Health Survey (*N* = 881 Communities).

		Mean	*SD*	Range
1	Prop. of ever-married women 15–49 years who were Muslim	.96	.10	0 to 1
2	Prop. of households in the top 40%th percentile on wealth	.52	.43	0 to 1
3	Prop. of ever-married women 15–49 years living in urban area	.51	.50	0 to 1
4	*Community FGMC Gender Norms Factor Score*	.00	1.0	-1.50 to 2.71
5	Prop. of ever-married women 20–49 years NOT cut	.11	.17	0 to 1
6	Prop. of ever-married women 20–49 years saying FGMC should STOP	.36	.24	0 to 1
7	Prop. of ever-married women 20–49 years saying religion does NOT require FGMC	.34	.22	0 to 1
8	*Community Extra-Familial Opportunities Factor Score*	.00	.93	-2.20 to 2.40
9	Prop. of ever-married women 20–49 years with age at first spousal coresidence 18+	.85	.13	0 to 1
10	Prop. of ever-married women 20–49 years married to a non-cousin	.68	.18	0 to 1
11	Prop. of ever-married women 20–49 years with secondary education or higher	.57	.24	0 to 1
12	Prop. of ever-married women 20–49 years working for cash or kind in the prior year	.16	.12	0 to 1

### Multilevel discrete-time hazard results

Results of multilevel discrete-time hazard models for a daughter’s risk of FGMC are in Table 3. Model 1, the unconditional model, includes dummy variables for daughter’s age risk sets. Model 2 adds level-one and level-two exposure variables. Model 3 adds hypothesized cross-level interactions. Model 4 adds level-one and level-two covariates and significant two-way interactions between age risk sets and covariates with non-proportional effects.

**Table 3:** Two-level discrete-time hazard models predicting daughter's risk of FGMC, 2014 Egypt Demographic and Health Survey (*N* = 14,171 mother-daughter pairs).

	Model 1		Model 2		Model 3		Model 4	
Included Variables	Coef	SE	Coef	SE	Coef	SE	Coef	SE
*Daughter Age Risk Set* (ref. 0 to 1)								
2 to 11	3.68***	.10	3.73***	.10	3.72***	.10	3.21***	.11
12 to 16	4.59***	.11	4.83***	.12	4.84***	.12	2.86***	.26
17 to 20	.34	.72	.76	.72	.77	.72	-.41	7.67
*Level-One Maternal Explanatory Variable*								
Opposition to FGMC (CM)			-1.48***	.06	-1.53***	.07	-0.82**	.26
*Level-Two Explanatory Variables*								
Comm. FGMC Gender Norms (GM)			-1.37***	.07	-1.44***	.08	-2.04***	.11
Comm. Extra-Familial Opportunities (GM)			-.04	.07	-.10	.07	.07	.11
*Cross-Level Interactions*: *Maternal Opposition to FGMC (CM)*								
x Comm. FGMC Gender Norms (GM)					-.17^†^	.10	-.29*	.12
x Comm. Extra-Familial Opportunities (GM)					-.32***	.09	-.41***	.11
*Level-One Daughter Covariates*								
Birth Year (GM)							-.03*	.02
Birth Order among Daughters (CM)							.17**	.05
*Level-One Maternal Covariates*								
Extra-Familial Opportunities (CM)							-.05	.08
Age in Years (GM)						.00		.01
Husband Secondary School or Higher (CM)							-.25***	.07
HH Wealth Index: Top 40% (CM; ref. Bottom 60%)						-.11		.12
*Level-Two Covariates*								
Proportion Living in an Urban Area (GM)							1.40***	.27
Proportion Muslim (GM)							-3.31***	.61
HH Wealth Index: Proportion in Top 40% (GM)							-1.68***	.38
*Age-Risk-Set Interactions*: *Maternal Opposition to FGMC (CM)*								
x Daughter age 2 to 11							-.76**	.27
x Daughter age 12 to 16							-1.52***	.30
x Daughter age 17 to 20						-2.81		2.27
*Age-Risk-Set Interactions*: *Daughter’s Birth Year (GM)*								
x Daughter age 2 to 11							-.33***	.02
x Daughter age 12 to 16							-.37***	.03
x Daughter age 17 to 20						-.13		.72
*Constant*	-5.62***	.11	-5.86***	.11	-5.91***	.11	-6.93***	.14
Level-Two residual variance, $\hat{\delta }$	2.06***	.16	1.06***	.09	1.06***	.09	2.07***	.18
Chi-Square	1728.02		2351.62		2336.17		2876.94	
AIC	13963.72		12785.87		12756.91		9657.34	
BIC	14005.08		12852.04		12839.63		9864.14	

***p ≤ .001

**p ≤ .01

* *p* ≤ .05

^†^ *p* ≤ .10

CM indicates that the variable is cluster-mean centered, and GM indicates grand-mean centered.

In Models 1–4, the logit hazard of FGMC among daughters was higher among 2–11- and 12–16-year-olds than among 0–1-year-olds (the reference group). The logit hazard was comparable between 17–20- and 0–1-year-olds. In unconditional models with single-year age dummies (available on request), the hazard of FGMC appeared to peak at age 12.

In Model 2, consistent with hypothesis 1 regarding direct community influences, community FGMC norms were negatively associated with the hazard that a daughter was cut. Namely, a daughter living in a community with less favorable FGMC norms among women had a lower logit hazard of experiencing FGMC. However, living in a community with more extra-familial opportunities for women had no significant direct association with the logit hazard that a daughter was cut. Stronger maternal opposition to FGMC (being uncut and having more unfavorable attitudes) was associated with a lower logit hazard of FGMC for her daughter.

In Model 3, both cross-level interactions were at least marginally significant, meaning the association of maternal opposition to FGMC with her daughter’s logit hazard of FGMC depended on community FGMC norms and extra-familial opportunities for women. In Model 4, with controls, associations of interest were robust, and sometimes larger, such as the interaction of community FGMC norms with maternal opposition. Thus, some covariates, such as the community proportions urban, Muslim, and with the wealthiest 40% of households, may be negative confounders.

**Fig 2:**
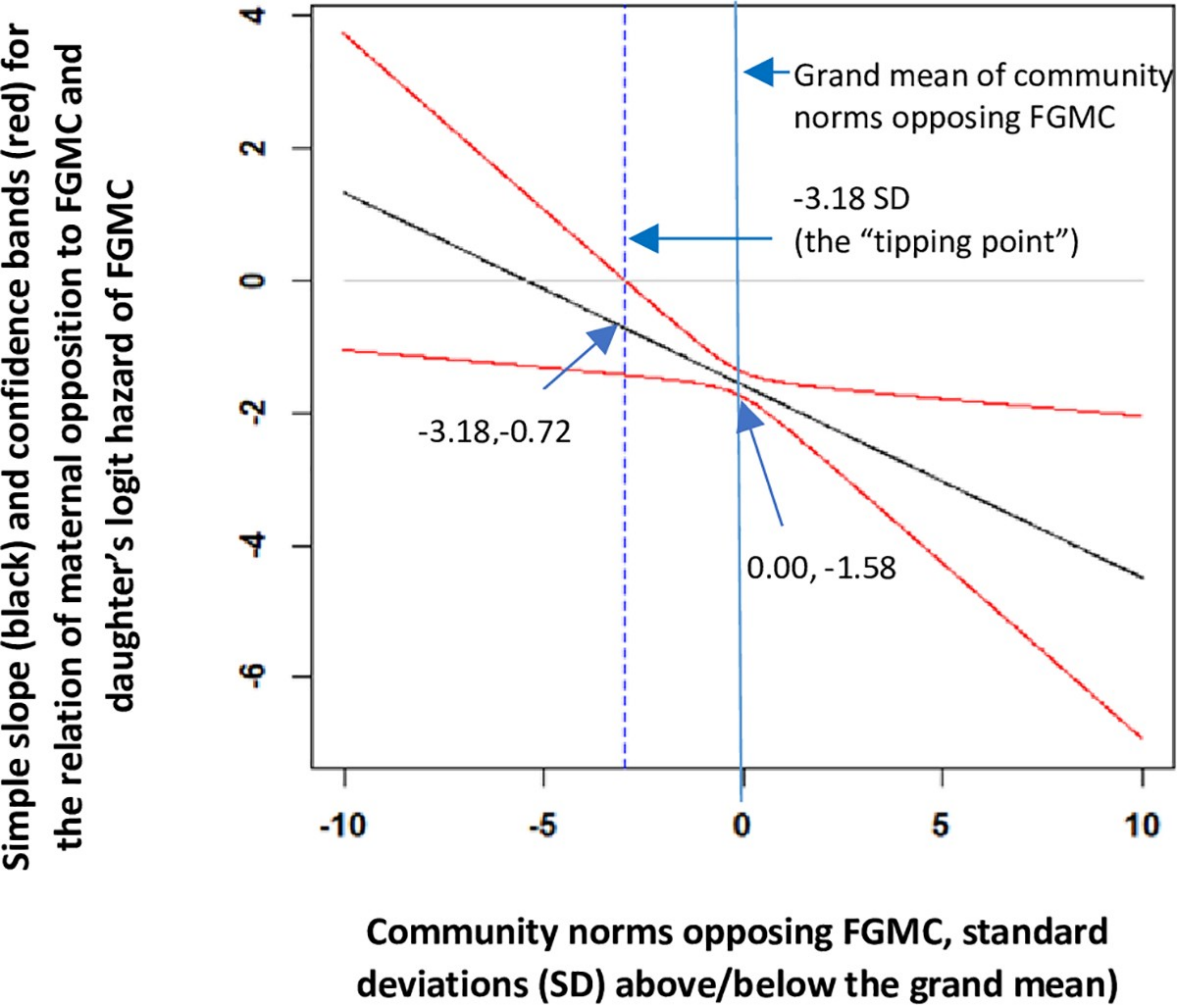
Predicted logit hazard of FGMC for the age-risk-set 2–11 years, Egypt 2014. Estimated logit hazards are generated from Table 3, Model 4 as a function of maternal opposition to FGMC at different levels of community norms opposing FGMC.

Based on Model 4, we graphed the simple slope (or estimated coefficient) and its 95% confidence bands for the relationship between a mother’s level of opposition to FGMC and her daughter’s predicted logit hazard of FGMC, conditional on the level of community opposition to FGMC (Fig 2). These estimates were generated for the age-risk-set 2–11, with the highest risk of cutting, while other covariates in the model were held constant at zero (i.e., the mean, since covariates were mean-centered). Fig 2 shows that the slope for maternal opposition to FGMC was more negative in communities with stronger opposition to FGMC. Specifically, maternal opposition to FGMC was more protective against a daughter’s risk of FGMC in communities that more strongly opposed FGMC. Using the online tool of Preacher and colleagues [93], we found that the relationship of maternal opposition to daughter’s FGMC was significant in communities where opposition to FGMC was 3.18 SD below the grand mean or higher (Fig 2). Thus, in communities where opposition to FGMC was *weakest* (3.18 SD below the grand mean or lower), maternal opposition to FGMC was unrelated to her daughter’s FGMC logit hazard, consistent with the idea that community opposition to FGMC must reach a “tipping point” before mothers who oppose FGMC enact their preferences. In this sample, however, only one community would have had a value less than 3.18 SD below the community grand mean of FGMC opposition.

**Fig 3:**
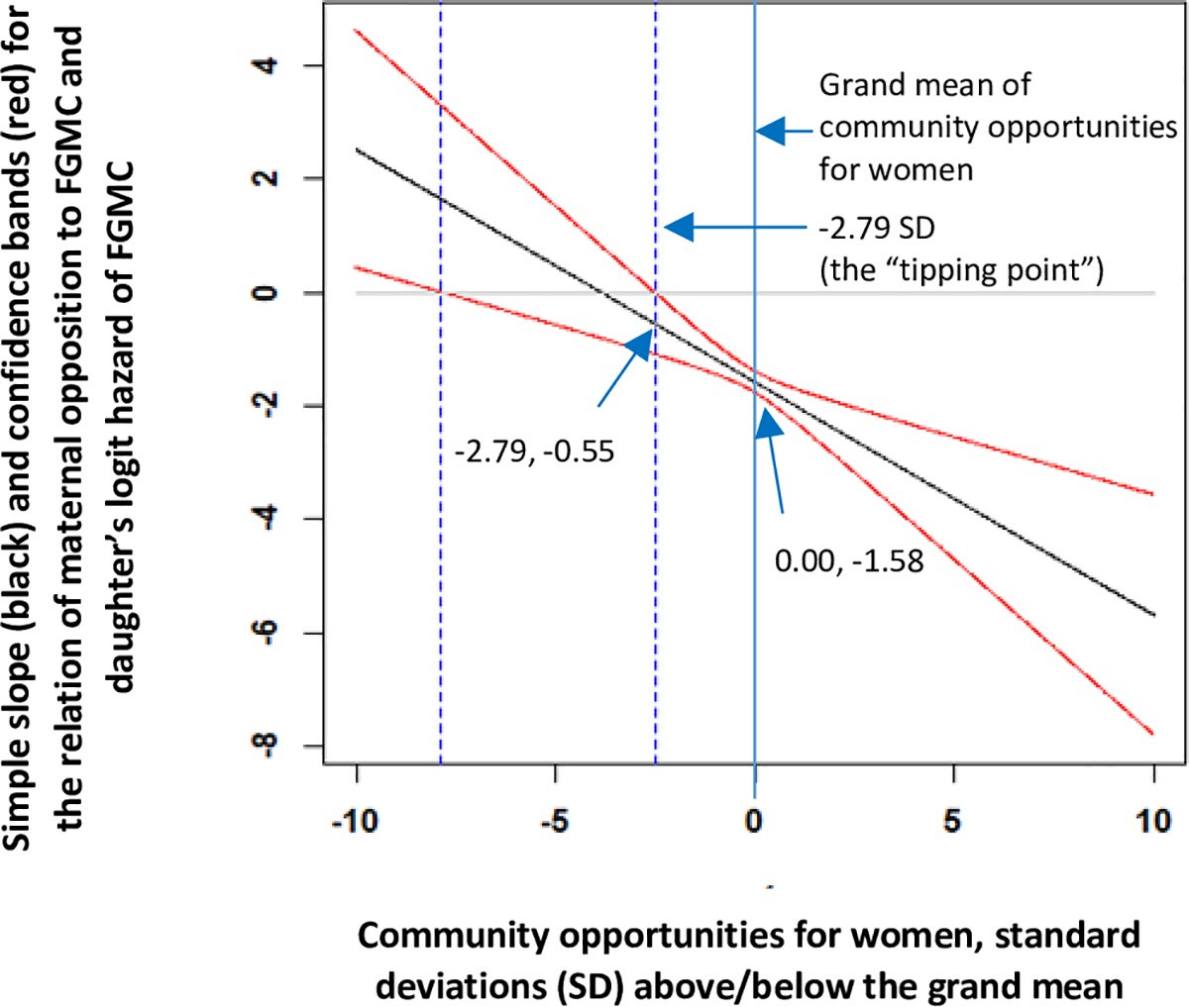
Predicted logit hazard of FGMC for the age-risk-set 2–11 years, Egypt 2014. Estimated logit hazards are generated from Table 3, Model 4 as a function of maternal opposition to FGMC at different levels of community opportunity for women outside the family.

Fig 3 shows a similar plot for the relationship of a mother’s level of opposition to FGMC with her daughter’s predicted hazard of FGMC across communities with different levels of opportunity for women, for the age-risk-set 2–11. The downward slope in the plot shows that the coefficient for maternal opposition to FGMC was more negative in communities with more opportunities for women. Specifically, maternal opposition to FGMC was more protective against a daughter’s risk of FGMC in communities where women had more opportunities outside the family. The coefficients for maternal opposition to FGMC were significant in communities with extra-familial opportunities 2.79 SD below the mean level of opportunity or higher. Thus, in addition to there being a “tipping point” of opposition to FGMC, as predicted by social norms theory, there appears to be a “tipping point” of women’s structural opportunity. In communities below this tipping point, mothers who oppose FGMC are less likely to enact their preferences. In this sample, however, only two communities would have a value less than 2.79 SD below the community grand mean of women’s opportunity. Results for the other age risk sets are consistent.

## Discussion

This analysis was the first to test an integrated gender-systems framework for the community-level influences on the hazard that a daughter experiences FGMC in a North African country that historically has adhered to a classic patriarchal family system [60]. We examined to what extent norms opposing FGMC and more structural opportunities for women outside the family were associated with a girl’s lower risk of FGMC. We also assessed to what extent maternal opposition to FGMC was more protective against her daughter’s hazard of cutting in communities more opposed to FGMC and having more extra-familial opportunities for women. We leveraged rich data on FGMC from the 2014 Egypt DHS, which is well-suited for multilevel analyses of community characteristics.

Certain descriptive findings are notable. Namely, 65% of 17–20-year-old daughters had experienced FGMC, compared to 93% of their mothers. These findings, given a peak age at FGMC of 12 years among daughters, are suggestive of substantial intergenerational change in the prevalence of FGMC. This dramatic intergenerational shift in the prevalence of FGMC makes our ecological approach and “tipping-point” hypothesis potentially more germane in the case of Egypt.

As expected, stronger community norms opposing FGMC were directly associated with a lower hazard that a daughter was cut (*H1*). These findings corroborate social-norms-based theories of FGMC [52, 53] and prior research showing strong associations between community prevalence of FGMC and a daughter’s hazard of cutting [19, 20]. Our conceptual framework extended norms-based theories and prior analyses by hypothesizing a direct relationship between community-level structural opportunities for women outside the family and a daughter’s hazard of FGMC. Contrary to our hypothesis, these opportunities for women were not directly associated with a daughter’s risk of cutting, net of individual maternal attitudes and community norms related to FGMC.

Our key conceptual innovation was the hypothesis that normative *and* structural characteristics of a *community gender system* condition the influence of maternal opposition to FGMC on her daughter’s FGMC risk. We found strong support for this hypothesis: maternal opposition to FGMC was more negatively associated with the hazard that her daughter was cut in communities with stronger norms opposing FGMC and greater extra-familial opportunities for women (*H2*). These findings were robust to the inclusion of controls, and therefore, provide good support for a gender-systems framework of the multilevel influences on FGMC. Together, our results illustrate the importance of considering structural and normative elements of gender systems to understand the persistence of FGMC at the village and neighborhood levels. Although women’s greater access to local opportunities outside the family was not directly associated with a daughter’s FGMC experience, such access appeared to promote mothers’ abilities to implement preferences against FGMC, either by increasing mothers’ decision-making power in the family or by changing their evaluation of future options for their daughters (or both). Our approach provides a potential model for extending norms-based theories to consider explicitly the role of local opportunity structures [42], as called for in recent reviews of norms-based health interventions [95].

Results were mixed about there being a “tipping point” in community opposition to FGMC and expansion of opportunities for women below which maternal opposition to FGMC has no influence. In most age risk sets, the coefficient for the relationship of maternal opposition to FGMC with a daughter’s risk of FGMC was significant and negative, even in communities that strongly favored FGMC and had limited opportunities for women, relative to the average community in Egypt. Evidence supporting a “tipping point” was strongest only for the youngest age risk set (0–1), in which the probability of FGMC was very low. It is possible that, even in communities with relatively unfavorable conditions for abandonment, the national context provides sufficient support to allow women who are personally opposed to FGMC some capacity to implement their preferences.

Egypt provides a salient test case for our framework, being the country with the largest number of girls and women who have been cut and a persistently high prevalence of FGMC, despite recent declines [13]. In some respects, our analyses in Egypt may offer a conservative test of our framework: countries having more variation in the practice of FGMC, and thus, a wider range of community normative and institutional contexts, might reveal an even stronger association between these aspects of a community gender system and a daughter’s risk of FGMC. Research is needed to understand how these associations may vary across countries. Egypt has a strong national identity with a large majority ethnic group and religion, although with visible minority groups [37]. Thus, the nature of social identity and meaning of FGMC for group belonging may differ in Egypt than in the multiethnic states that dominate sub-Saharan Africa. Moreover, although a large rural population exists in Egypt, the Egyptian economy is more diversified and less dependent on subsistence agriculture than are the economies of other countries where FGMC is common. The greater economic opportunity outside family-based agriculture, alongside persistent elements of classic patriarchy that reinforce women’s economic dependence on male family members [60], may make expanded opportunities for women outside the family more consequential in Egypt than elsewhere [38]. Also, conditions surrounding the practice of FGMC in diaspora communities, where the maintenance of gender norms is tied to questions of cultural preservation and immigrant integration, may require specific theorization.

### Implications for policies and programs

Our findings have several implications for policies and programs. Community-level opposition to FGMC had a direct association with daughters’ FGMC risk and strengthened the protective effect of maternal opposition to FGMC. This finding corroborates evidence from TOSTAN, a quasi-experimental intervention study of the relationship between a human-rights-based community empowerment and educational curriculum and girls’ risk of experiencing FGMC. In TOSTAN, communities were engaged in participatory dialogues and community-based activities grounded in the principles of human rights, democratic processes, and women’s health, among other topics. At the completion of these dialogues, communities deliberated and spearheaded collective decisions about abandoning harmful practices, including FGMC, and engaged neighboring communities to adopt the same new norms. The study showed evidence of diffusion of abandonment from program participants to non-participants and more unfavorable attitudes about FGMC and lower prevalence of cutting girls in intervention than comparison areas [96]. Thus, interventions aimed at changing collective perceptions of FGMC—such as community pledges to reject FGMC—may have multiplicative impacts on daughters’ outcomes.

Community-level opportunities for women were not directly associated with daughters’ FGMC risk, but our results imply that improving opportunities for women may reduce the risk of FGMC among daughters whose mothers oppose the practice. Thus, future community-based interventions might consider joint activities that involve educational opportunities and training in income-generating activities for women. Future research also should explore the joint influence of community FGMC norms among men on daughters’ risk of FGMC and whether engaging men in community-based programs would help to accelerate the abandonment of FGMC. Going forward, practitioners should consider intervention packages that address community gender norms specific to FGMC and that provide enabling resources to women, which empower mothers collectively to imagine alternatives to dependence on marriage in the life course trajectories of their daughters. Any effort to shift gender norms and opportunity structures will be most effective if it attempts to respect the values of local communities.

Despite the importance of community context, we also found that maternal opposition to FGMC was protective for daughters at most ages, even in communities that strongly opposed change. Thus, even where community-level interventions are infeasible, interventions focused on the beliefs of individuals may support change. Individual-level interventions might include provider support for non-cutting during clinic visits, or one-on-one support from outreach workers or religious leaders.

### Limitations and implications for research

The limitations of this analysis inform recommendations for research. First, the cross-sectional design of the DHS limited our ability to establish the timing of community-level characteristics before the onset of a daughter’s risk of cutting. We addressed this limitation by constructing community-level exposures using data from ever-married women 20–49 years, an older cohort than the one for which the FGMC outcome was observed. Still, if the composition of a community changed substantially or if families moved between a daughter’s birth and the survey, our community-level measures may have misrepresented the salient factors at the time of decision-making about FGMC. This measurement error would tend to reduce the precision of estimates and downwardly bias coefficients. Re-estimating final models without the oldest daughters (15–20 years) confirmed that the relationship of community FGMC norms with a daughter’s risk of FGMC was stronger. Thus, longitudinal surveys are needed to measure time-varying community characteristics before the onset of a daughter’s risk of FGMC.

Second, the DHS sample cluster proxied for a village or neighborhood “community.” The DHS is not designed for aggregation at the cluster level, and sample averages may produce imprecise estimates of true cluster characteristics, especially when the number of women per cluster is small and the intra-class correlation (ICCs) of the predictors is low [97]. Depending on the characteristic being estimated, sample means also may produce biased estimates if the degree of precision is related to other community characteristics. In this sample, the mean cluster size was 24 ever-married women 20–49 years (range 1–127, SD 13). Estimated ICCs for community opportunities and community norms were .30 and .27, respectively, above the .20-level at which bias becomes unacceptable for the standard DHS cluster size of 25 respondents [97]. Still, the average cluster size was small, so we cannot state definitively the level of the bias of cluster-level estimates without conducting simulations. Prior simulations have shown that the bias produced by using DHS sample clusters to estimate community characteristics is small [97]. Surveys intended for multilevel analyses should draw, within villages or neighborhoods, separate samples that are large enough to construct unbiased estimates of community-level characteristics that are measured by aggregating individual responses [98].

Third, a conceptual concern with using the DHS sample cluster as the measure of community is that the cluster may not capture the multiple social communities in which mother-daughter pairs are embedded [43]. Network, spatial, and qualitative data to measure women’s overlapping activity spaces and social-group memberships would offer a more nuanced understanding of the relevant socio-contextual influences on a daughter’s risk of experiencing FGMC.

Fourth, our measure for women’s community-level structural opportunities outside the family relied on indicators of schooling attainment, engagement in market work, and age at first residence with a spouse. These measures are important but may not capture the full range of women’s local opportunities, nor women’s experience of opportunity. More contextualized community-level measures of women’s opportunities, such as those related to their political participation or empowerment, may influence more strongly a daughter’s risk of FGMC.

Fifth, the community-level exposures of interest were correlated, such that communities tending to oppose FGMC also tended to have more opportunities for women outside the family. Our gender-systems framework predicted some correlation among these measures and their distinct influences; still, distortion due to multi-collinearity may be a concern. In descriptive analyses, these community-level exposures had an estimated Pearson pairwise correlation of 0.64; however, in models using only one of the two community-level exposures, standard errors for the two community measures changed little in these models, suggesting that collinearity was not a major problem. Thus, these constructs arguably were correlated but distinct components of a community gender system.

Finally, our analysis focused on two levels–the mother-daughter pair and the community. Yet, the influences of regional or national conditions warrant consideration. In sensitivity analyses, results for primary explanatory variables were robust to including region fixed effects in our final model. Since our primary interest was in community norms, community opportunity structures, and cross-level interactions, we retained the more parsimonious model without regional dummies. In future analyses, comparing individual, community, and national influences on a daughter’s FGMC risk would reflect a comprehensive ecological model of change.

### Conclusion

Results provide good support for a gender-systems framework of the multilevel influences on FGMC. Integrated, multilevel interventions that address community-level gender norms about FGMC, community-level opportunities for women outside the family, and personal beliefs about FGMC among mothers of at-risk daughters may be needed for sustainable declines in the practice. Further conceptual and empirical work is needed to understand the broader role of opportunity structures for women in the relationship of gender norms to outcomes for girls.
